# Genetic prevalence and clinical relevance of canine Mendelian disease variants in over one million dogs

**DOI:** 10.1371/journal.pgen.1010651

**Published:** 2023-02-27

**Authors:** Jonas Donner, Jamie Freyer, Stephen Davison, Heidi Anderson, Matthew Blades, Leena Honkanen, Laura Inman, Casey A. Brookhart-Knox, Annette Louviere, Oliver P. Forman, Rebecca Chodroff Foran

**Affiliations:** 1 Wisdom Panel Research Team, Wisdom Panel, Kinship, Helsinki, Finland; 2 Wisdom Panel Research Team, Wisdom Panel, Kinship, Portland, Oregon, United States of America; 3 Wisdom Panel Research Team, Wisdom Panel, Kinship, Leicestershire, United Kingdom; University of Bern, SWITZERLAND

## Abstract

Hundreds of genetic variants implicated in Mendelian disease have been characterized in dogs and commercial screening is being offered for most of them worldwide. There is typically limited information available regarding the broader population frequency of variants and uncertainty regarding their functional and clinical impact in ancestry backgrounds beyond the discovery breed. Genetic panel screening of disease-associated variants, commercially offered directly to the consumer or via a veterinary clinician, provides an opportunity to establish large-scale cohorts with phenotype data available to address open questions related to variant prevalence and relevance. We screened the largest canine cohort examined in a single study to date (1,054,293 representative dogs from our existing cohort of 3.5 million; a total of 811,628 mixed breed dogs and 242,665 purebreds from more than 150 countries) to examine the prevalence and distribution of a total of 250 genetic disease-associated variants in the general population. Electronic medical records from veterinary clinics were available for 43.5% of the genotyped dogs, enabling the clinical impact of variants to be investigated. We provide detailed frequencies for all tested variants across breeds and find that 57% of dogs carry at least one copy of a studied Mendelian disease-associated variant. Focusing on a subset of variants, we provide evidence of full penetrance for 10 variants, and plausible evidence for clinical significance of 22 variants, on diverse breed backgrounds. Specifically, we report that inherited hypocatalasia is a notable oral health condition, confirm that factor VII deficiency presents as subclinical bleeding propensity and verify two genetic causes of reduced leg length. We further assess genome-wide heterozygosity levels in over 100 breeds, and show that a reduction in genome-wide heterozygosity is associated with an increased Mendelian disease variant load. The accumulated knowledge represents a resource to guide discussions on genetic test relevance by breed.

## Author summary

Hundreds of genetic mutations associated with risk for disease have been identified in dogs and genetic testing is offered for most of them. Accurate information about which mutations are common and which mutations cause disease regardless of the breed or ancestry of a dog are critical for the ability to make informed pet care treatment choices and sustainable breeding selections. We examined the largest canine cohort examined in a single study to date, consisting of around one million dogs from our existing cohort of more than 3.5 million dogs. The dogs were screened for 250 genetic variants to shed light on their prevalence and breed distribution. We used electronic medical records from veterinary clinics to establish the relevance of specific genetic risk factors for pet care. We found that disease-associated variants are collectively common in dogs and that many health issues are shared by both mixed breed and purebred dogs. Our study also emphasizes the importance of avoiding excessive inbreeding in dogs by associating lower genetic diversity levels with an increased risk for the manifestation of inherited diseases. Taken together, our study provides an important resource to guide genetic counseling for inherited diseases across the dog population.

## Introduction

The domestic dog has been established as a favorite study model for geneticists due to its population structure, genomic architecture, and human comparable phenotypes that enable research to understand the molecular background of inherited diseases and their treatments [1, 2]. Such discoveries can lead to advancements that benefit human health, while serving the dog breeder and veterinary communities through the availability of genetic tests that can help to guide breeding selection and veterinary care. With more than 300 canine Mendelian disease and trait variants identified to date [3], many of which form the basis of commercially available genetic tests, it has become increasingly challenging for breeders, breed health advisors, kennel clubs and breed registries, veterinary clinicians, and scientists to stay informed about which genetic tests are relevant for which breeds and to determine the level of population, functional, and clinical evidence supporting the use of each test. Among the main factors shaping the disease variant heritage of the purebred dog population are breed formation, or founding, events that are typically coupled with closing of the breed registry to limit additional gene flow to the novel breed. Later crossbreeding can transfer disease variants from one breed to another as we have shown earlier [4], while mutations arising within breeds further shape the gene pool. While the presence or absence of a disease variant in a specific breed background can be understood in the context of breeding practices, their full distribution across breeds remains elusive without extensive genetic screening in large and diverse sample sets. There are commendable initiatives to compile and make information available on inherited diseases and traits, genetic variants, genetic test availability, and genetic test relevance in dogs (e.g., [3, 5, 6]). Additional ongoing large scale canine projects are aiming to collect phenotype information through veterinary records or community science efforts to uncover the frequency of health issues and understand the genetic and environmental factors influencing disease, behavior, and aging (e.g., [7–9]). However, one constraint of the currently available resources is the limited availability of follow-up, or routinely updated information on the frequency of genetic variants compiled after the original research discovery publication. Other current knowledge gaps related to canine disease-associated variants stem from discovery studies published without conclusive functional evidence supporting candidate variant causality and from the limited opportunities of original studies to explore variant impact on a broad variety of breed backgrounds beyond the initial discovery breed.

Canine health screening schemes for breeding animals such as hip scoring and ophthalmoscopic examinations have been in use for decades to combat hereditary disease (e.g., [10, 11]), and such programs remain valuable today especially for multifactorial diseases and when the molecular genetic background of a condition is not fully understood. Multiplex or panel screening of known genetic disease variants has emerged as a technologically feasible, cost-efficient, and scientifically justified complementary approach to simultaneously screen any individual dog for a large number of disease-associated variants [4, 12]. For the first time ever, multiplex screening has recently been introduced on a large scale into the veterinary clinical setting through the inclusion of routine DNA testing in pet care plans and electronic medical records (EMRs) across more than a thousand clinics in the United States [13]. Together with large-scale health surveys, this development allows for the establishment of cohorts with deep phenotypic and genetic information that hold great potential for addressing some of the knowledge gaps related to not only the genetic prevalence and distribution of disease-associated variants in the population, but also to the clinical relevance of specific genetic testing results.

Genetic test results for Mendelian disease variants should also be considered in the broader context of population genetic diversity and the viability of the gene pool. One mechanism in which inbreeding depression (reduced survival and fitness in the offspring of close relatives) can act is through the accumulation of deleterious recessive mutations that negatively impact the health and fertility/fecundity of the individual [14]. The link between genomic inbreeding levels and homozygosity for known disease variants has so far not been exhaustively explored across dog breeds with direct simultaneous genetic measurement of both variables in the same individual, although considerable overlap between regions of homozygosity (ROH) and “at risk” genotypes at 29 recessive disease loci was previously reported [15]. Molecular estimates of genomic inbreeding levels for breed comparisons can be obtained through genotyping single nucleotide polymorphisms (SNPs), for example through array-based approaches, enabling simultaneous screening for genetic diseases, while providing a more accurate representation of genetic population measurements of diversity compared to pedigree-based metrics [16].

We have previously explored canine genetic disease prevalence through analyses of 152 Mendelian disease-associated variants in around 100,000 dogs [12]. In the current study, we aim to expand our work by genotyping a separate cohort consisting of 1,054,293 dogs for 250 genetic variants previously associated with canine disease to further understand their frequency and distribution in the general canine population. Moreover, we seek to obtain additional evidence supporting the association between the variants and the respective phenotypes to which they have previously been linked using follow-up studies based on electronic medical records (EMRs) and pet owner interviews. We additionally explore the relationship between genome-wide inbreeding level and Mendelian disease variant load. We have previously shown that a higher proportion of mixed breed dogs are heterozygous for common recessive disease-associated variants compared to purebred dogs, while conversely a higher proportion of purebred dogs are homozygous for the same recessive variants compared to mixed breed dogs [12]. In the present study, we further refine understanding of this pattern by leveraging the availability of genome-wide SNP-based heterozygosity estimates for individual dogs of the studied cohort, acquired as a part of commercial genetic testing.

## Results

### Quantitative analysis highlights the ubiquitous nature of canine disease-associated variants

**Fig 1:**
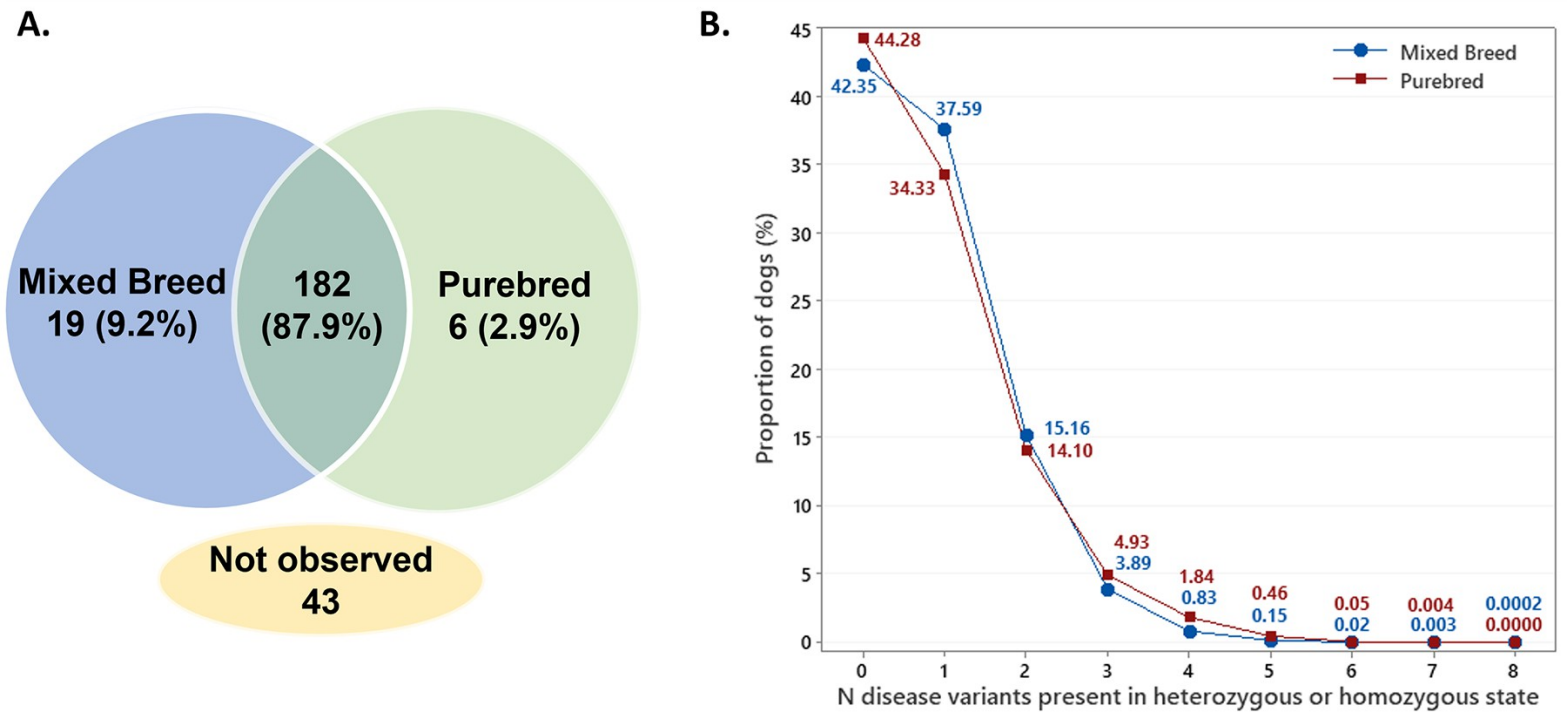
A) Presence of 250 Mendelian disease-associated variants and B) distribution of 242 Mendelian disease-associated variants in a cohort of 811,628 mixed breed and 242,665 purebred dogs.

Of the 250 genetic variants following Mendelian inheritance patterns and selected for screening based on their previously implicated involvement in canine inherited disorders, 207 (82.8%) were observed in a minimum of one dog in the study population of 1,054,293 dogs. The majority of the observed disease-associated variants (N = 182; 87.9% of all observed disease-associated variants) were encountered in both mixed breed and purebred dogs while 19 variants (9.2%) were exclusive to the mixed breed population and 6 (2.9%) to the purebred population (Fig 1A and S1 Table). The maximum number of screened disease-associated variants present in any one dog was eight, which was observed in two mixed breed dogs. Restricting the investigation to the subset of 242 variants (S1 Table) genotyped in the full cohort, we observed that 57.2% of all dogs carried at least one of the tested disease-associated variants in either heterozygous or homozygous state (Fig 1B).

### Decrease in genome-wide heterozygosity is associated with an increased Mendelian disease variant load

**Fig 2:**
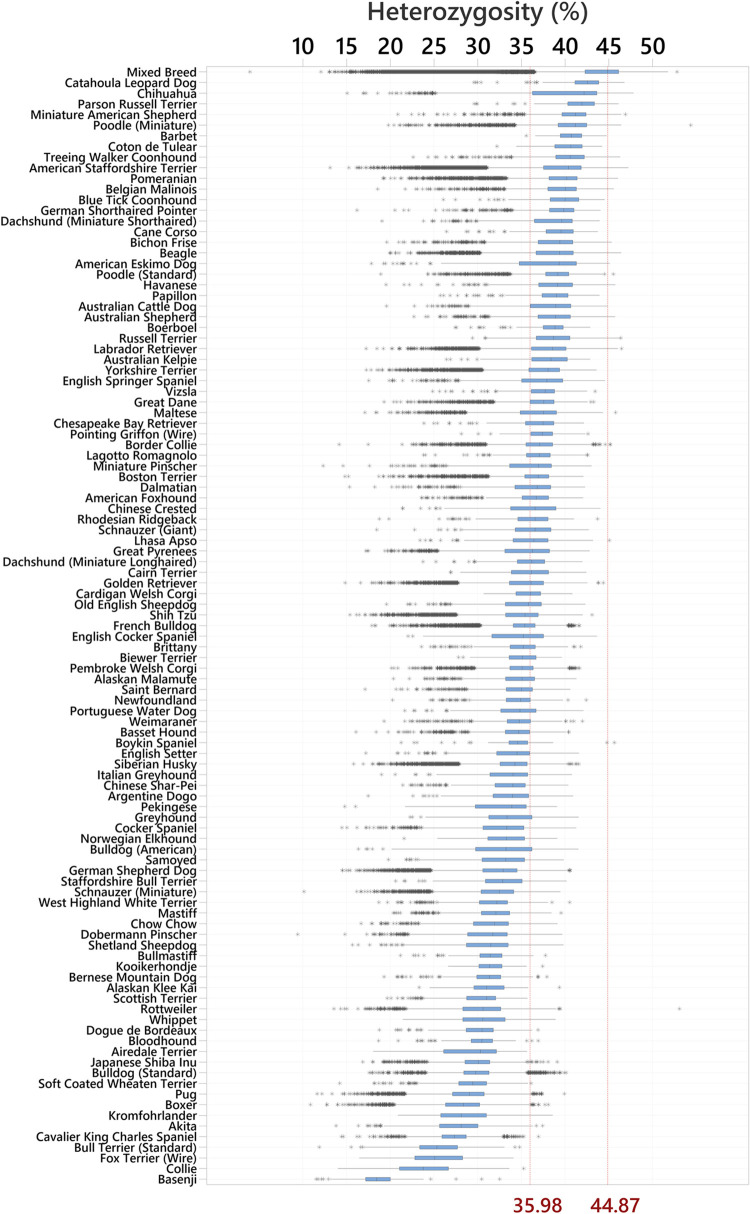
Genome-wide heterozygosity in mixed breed dogs and 104 breeds (N ≥100 dogs tested) ranked from most to least diverse. The figure shows medians and interquartile range boxes representing the middle 50% of the data, with whiskers representing the ranges for the bottom and top 25% of the data values excluding outliers (data points >1.5x the interquartile range from the boxes; shown as asterisks). The red vertical reference lines represent the median heterozygosity in the combined purebred dog group (35.98%) and mixed breed dogs (44.87%).

Genome-wide SNP-based heterozygosity estimates were acquired in conjunction with the disease variant genotyping and were routinely available for dogs of the screened cohort. As expected, we found that purebred dogs (N = 242,665) had a lower mean heterozygosity level than mixed breed dogs (N = 811,628), indicating a higher mean level of genetic inbreeding in purebreds (35.38% [95% confidence interval (CI) 35.36, 35.40] vs 43.15% [95% CI 43.14, 43.16] heterozygous genotyped SNP loci respectively; Mann-Whitney W = 5.03x10^11^, P < 10^−16^), and we considered these two groups separately in subsequent analyses. Fig 2 further details heterozygosity levels by breed for the 104 breed groups that were represented by ≥100 individuals while full statistics for all breeds are available in S2 Table and heterozygosity values for all individuals at https://doi.org/10.5061/dryad.tdz08kq3j.

**Fig 3:**
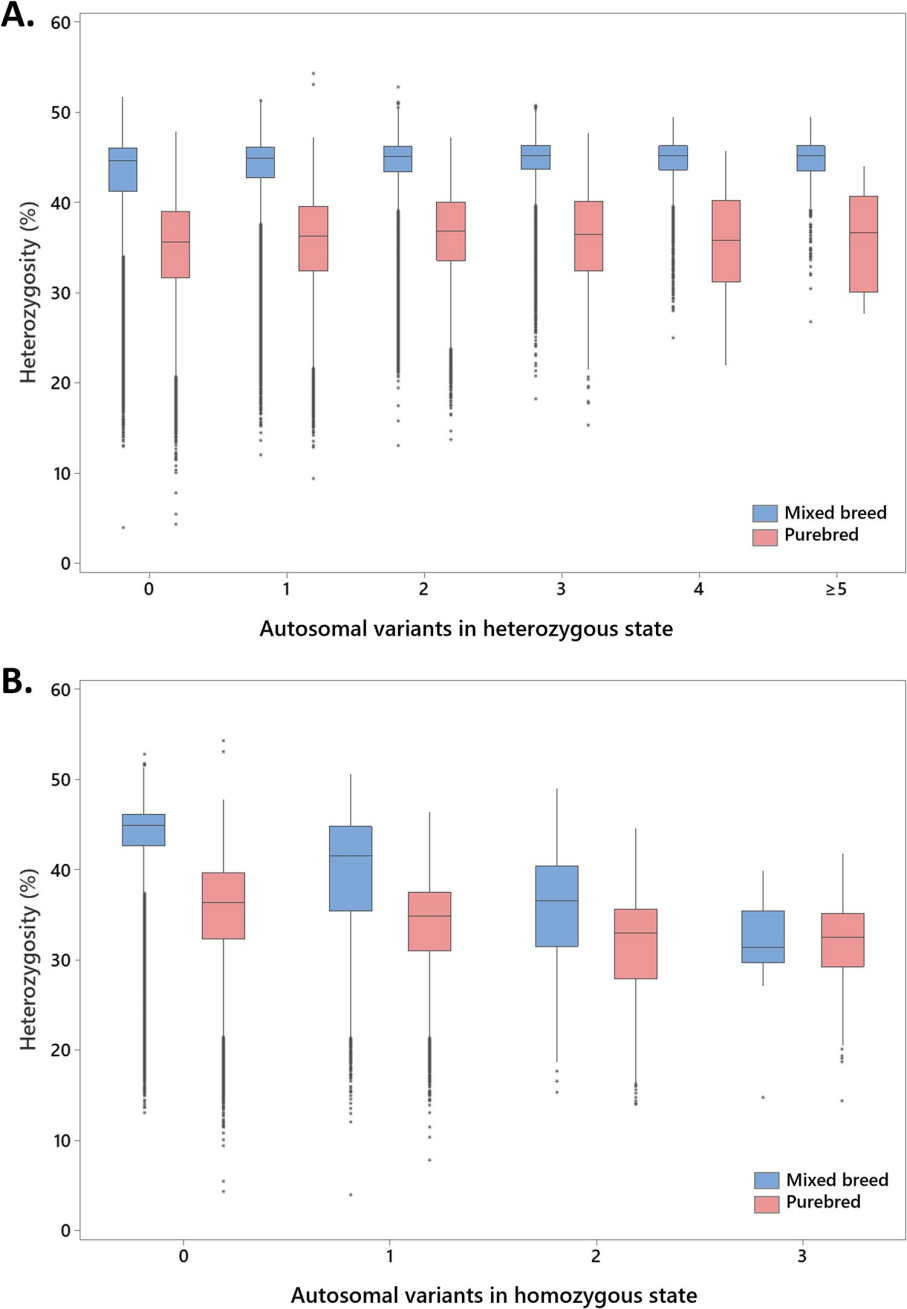
The relationship between genome-wide genetic heterozygosity and autosomal disease-associated variants present in A) heterozygous state and B) homozygous state; based on 178 recessive and 13 semi-dominant or dominant variants tested in 1,054,293 dogs. Medians and interquartile range boxes representing the middle 50% and whiskers representing the bottom and top 25% of the data excluding outliers (data points >1.5x the interquartile range from the boxes; asterisks) are shown.

We examined the correlation between genome-wide levels of genetic diversity and genotypes for autosomal disease-associated variants (178 variants following recessive and 13 semi-dominant or dominant modes of inheritance; S1 Table) present and genotyped in the full study sample. We found that increased genome-wide genetic heterozygosity was weakly, but significantly, correlated with the presence of an increased number of autosomal disease-associated variants in heterozygous state in both mixed (Spearman r = 0.082, P < 0.001) and purebred dogs (Spearman r = 0.082, P < 0.001; Fig 3A). Conversely and more notably, we found that a decrease in heterozygosity manifests as an increased number of homozygous autosomal disease-associated variants (Fig 3B). This pattern was observed in both dogs classified as mixed breed (r = -0.16, P < 0.001) and purebred (r = -0.136, P < 0.001) and persisted when the analysis was repeated including only the recessive disease-associated variants (mixed breed r = -0.118, P < 0.001 and purebreds r = -0.155, P < 0.0001).

### The most common disease-associated variants in dogs

The top 20% of the most prevalent disease alleles collectively accounted for 98.3% of all disease-associated alleles observed in the study sample, and the distribution was highly similar across both the mixed breed dogs and combined purebred sample groups (Table 1; full details provided as S1 Table). Only 7 of the 50 most common variants in mixed breed dogs were not present among the purebred top 50 variants and vice versa. Among the most common variants were several variants with a suggested ancient origin and established distribution across a wide range of modern-day breeds, such as chondrodystrophy and intervertebral disc disease (CDDY), degenerative myelopathy (DM), progressive rod-cone degeneration (prcd-PRA), hyperuricosuria (HUU), and collie eye anomaly (CEA) [17–21]. We also observed several variants that had only previously been associated with disease in a single breed or sub-population, at high frequency across many breeds. This includes the *IGFBP5* gene candidate variant for bald thigh syndrome in sighthounds [22], *SLC7A9* and *SLC3A1* gene variants associated with cystinuria in Bulldogs [23, 24], as well as *PDK4* and *TTN* gene risk variants for dilated cardiomyopathy (DCM) in Dobermans [25–27]. The clinical impact of these broadly distributed putative genetic risk factors in dogs of a diverse genetic ancestry background were further assessed with the use of electronic medical records.

**Table 1:** Putative disease-associated variants with allele frequency >0.5% in the examined canine population.

OMIA variant ID^1^	Variant phenotype	Mode of inheritance	Gene	Variant^2^	Allele frequency all dogs [%]	Allele frequency mixed breed dogs [%]	Allele frequency purebred dogs [%]
855	Chondrodystrophy and Intervertebral Disc Disease Risk (CDDY)	Autosomal dominant	*FGF4*	chr12:33710178ins	11.951	11.353	13.945
36	Degenerative Myelopathy (DM)	Autosomal recessive (Incomplete penetrance)	*SOD1*	chr31:26540342G>A	7.858	7.506	9.037
699	Cone-Rod Dystrophy (cord1-PRA/crd4)	Autosomal recessive (Incomplete penetrance)	*RPGRIP1*	chr15:18332036_18332037ins[A [29];GGAAGCAACAGGATG]	3.809	3.641	4.370
76	Progressive Rod-Cone Degeneration (prcd-PRA)	Autosomal recessive	*PRCD*	chr9:4188663C>T	2.923	3.314	1.613
[22]	Bald Thigh Syndrome (Discovered in Sighthounds)	Autosomal recessive	*IGFBP5*	chr37:23582195C>T	2.373	2.742	1.358
[23]	Cystinuria Type I-B (*SLC7A9* p.A217T)	Autosomal recessive (Incomplete penetrance)	*SLC7A9*	chr1:119209134G>A	1.991	1.603	3.291
83	Hyperuricosuria (HUU)	Autosomal recessive	*SLC2A9*	chr3:69456869G>T	1.873	1.841	1.982
[23]	Cystinuria Type I-A (*SLC3A1* p.I192V)	Autosomal recessive (Incomplete penetrance)	*SLC3A1*	chr10:46705989A>G	1.739	1.372	2.969
[23]	Cystinuria Type I-A (*SLC3A1* p.S698G)	Autosomal recessive (Incomplete penetrance)	*SLC3A1*	chr10:46735617A>G	1.516	1.118	2.847
[25]	Dilated Cardiomyopathy risk factor (Discovered in the Doberman Pinscher; *PDK4*-related)	Autosomal dominant (Incomplete penetrance)	*PDK4*	chr14:20829684_20829699del	1.272	1.258	1.321
632	Collie Eye Anomaly (CEA)	Autosomal recessive	*NHEJ1*	chr37:25698028_25705826del	1.083	1.141	0.888
39	Exercise-Induced Collapse (EIC)	Autosomal recessive (Incomplete penetrance)	*DNM1*	chr9:55282762C>A	0.951	0.923	1.046
[26]	Dilated Cardiomyopathy risk factor (Discovered in the Doberman Pinscher; *TTN*-related)	Autosomal dominant (Incomplete penetrance)	*TTN*	chr36:22321955C>T	0.947	1.022	0.698
469	MDR1 (Multidrug Resistance 1) Medication Sensitivity	Autosomal dominant	*ABCB1*	chr14:13726596_13726599del	0.926	0.974	0.763
616	Ichthyosis (Discovered in the Golden Retriever)	Autosomal recessive	*PNPLA1*	chr12:5417388_5417390delinsTACTACTA	0.910	0.734	1.500
401	Von Willebrand’s Disease, Type 1 (vWD 1)	Autosomal recessive	*VWF*	chr27:38951839G>A	0.776	0.736	0.913
275	Canine Multifocal Retinopathy 1 (Discovered in Mastiff-related breeds; CMR1)	Autosomal recessive	*BEST1*	chr18:54478586G>A	0.701	0.607	1.013
1050	Stargardt Disease (Discovered in the Labrador Retriever)	Autosomal recessive	*ABCA4*	chr6:55146556dup	0.626	0.576	0.791

^1^ A reference is listed where a specific OMIA (Online Mendelian Inheritance in Animals; omia.org) variant ID was not available at the time of writing

^2^ CanFam3.1 coordinates

### Uncovering the breed distribution of canine genetic variants

The detailed breed distributions and allele frequencies of all variants tested in the present study, including the aforementioned high frequency variants in the *IGFBP5*, *SLC7A9*, *SLC3A1*, *PDK4* and *TTN* genes, are summarized and available as a resource for the community in S3 Table while the raw genotype dataset is available at https://doi.org/10.5061/dryad.tdz08kq3j [28]. For the purposes of this study, we defined a novel breed finding of particular interest as a disease-associated variant present in an additional pure breed with an allele frequency of ≥1%, with the additional requirement of more than one alternative allele heterozygous or homozygous dog observed. With this definition, we discovered the genetic presence of 26 disease-associated variants in a total of 65 pure breeds in which they, to the best of our knowledge, have not been characterized previously in the peer-reviewed scientific literature (Table 2).

**Table 2:** Disease-associated variants found in additional breeds at an allele frequency ≥1%

OMIA Variant ID^1^	Variant phenotype	Mode of inheritance	Gene	Variant^2^	Previously identified breed(s)	Additional identified breeds (Total N dogs screened; Variant frequency)
444	Acral Mutilation Syndrome (AMS)	Autosomal recessive	*GDNF*	chr4:70875561C>T	English Pointer, English Springer Spaniel, French Spaniel, German Shorthaired Pointer	Cirneco dell’Etna (7; 14.29%) Irish Terrier (35; 2.86%) Old English Sheepdog (423; 7.92%)
1044	Amelogenesis Imperfecta (Discovered in the Parson Russell Terrier; AI)	Autosomal recessive	*ENAM*	chr13:59945218C>T	Parson Russell Terrier	Danish Swedish Farmdog (61; 3.28%)
275	Canine Multifocal Retinopathy 1 (Discovered in Mastiff-related breeds; CMR1)	Autosomal recessive	*BEST1*	chr18:54478586G>A	Boerboel, Bull Mastiff, English Mastiff, Great Pyrenees	Boston Terrier (3702; 7.47%) Neapolitan Mastiff (90; 1.11%)
422	Canine Scott Syndrome (CSS)	Autosomal recessive	*ANO6*	chr27:8912219C>T	German Shepherd Dog	Presa Canario (64; 1.56%)
632	Collie Eye Anomaly (CEA)	Autosomal recessive	*NHEJ1*	chr37:25698028_25705826del	>10	Anatolian Shepherd Dog (66; 3.79%) Lacy Dog (32; 9.38%) Maremma Sheepdog (37; 2.7%) McNab (28; 17.86%)
699	Cone-Rod Dystrophy (cord1-PRA/crd4)	Autosomal recessive (Incomplete penetrance)	*RPGRIP1*	chr15:18332036_18332037ins[A [29];GGAAGCAACAGGATG]	Miniature Long-haired Dachshund	Airedale Terrier (200; 3.5%) American Eskimo Dog (302; 3.48%) American Staffordshire Terrier (42793; 17.75%) Australian Shepherd (2296; 1.57%) Barbet (106; 5.66%) Basset Hound (990; 1.32%) Beagle (5292; 10.51%) Bedlington Terrier (11; 9.09%) Bloodhound (280; 21.79%) Catahoula Leopard Dog (154; 4.25%) Caucasian Shepherd Dog (41; 2.44%) Chihuahua (4273; 3.88%) Clumber Spaniel (12; 37.5%) English Cocker Spaniel (580; 1.73%) Field Spaniel (29; 12.07%) French Bulldog (13114; 7.96%) Kai Ken (9; 33.33%) Lagotto Romagnolo (623; 4.27%) Poodle (Miniature) (3555; 1.13%) Pumi (86; 4.65%) Rottweiler (4718; 6.82%) Schnauzer (Standard) (75; 12.67%) Silky Terrier (28; 8.93%) Weimaraner (647; 6.66%)
1037	Congenital Dyshormonogenic Hypothyroidism with Goiter (Discovered in the Shih Tzu)	Autosomal recessive	*SLC5A5*	chr20:45024672C>T	Shih-Tzu	Pekingese (239; 1.05%)
1079	Deafness and Vestibular Dysfunction (Discovered in the Doberman Pinscher)	Autosomal recessive	*MYO7A*	chr21:21563111C>T	Doberman Pinscher	Mudi (42; 4.76%)
[46]	Ehlers-Danlos Syndrome (Discovered in the Chihuahua and Poodle)	Autosomal recessive	*TNXB*	chr12:1490385G>A	Chihuahua, Poodle	German Pinscher (13; 38.46%) German Shorthaired Pointer (65; 2.31%) Pomeranian (121; 4.55%)
39	Exercise-Induced Collapse (EIC)	Autosomal recessive (Incomplete penetrance)	*DNM1*	chr9:55282762C>A	Chesapeake Bay Retriever, Curly-coated retriever, Labrador Retriever	Maltese (2413; 2.15%)
40	Factor VII Deficiency	Autosomal recessive	*F7*	chr22:60578895G>A	>25	Italian Greyhound (263; 1.33%) Lacy Dog (32; 10.94%) Schipperke (72; 9.03%)
970	Hereditary Nasal Parakeratosis (Discovered in the Greyhound; HNPK)	Autosomal recessive	*SUV39H2*	chr2:21731812_21731815del	Greyhound	Saluki (24; 4.17%)
83	Hyperuricosuria (HUU)	Autosomal recessive	*SLC2A9*	chr3:69456869G>T	Dalmatian	Argentine Dogo (225; 2%) Catahoula Leopard Dog (154; 1.95%) German Wirehaired Pointer (84; 1.79%) Munsterlander (Small) (15; 10%)
49	Hypocatalasia	Autosomal recessive	*CAT*	chr18:33397548C>T	American Foxhound, Beagle	Plott (25; 8%)
98	Macrothrombocytopenia (Discovered in the Norfolk and Cairn Terrier)	Autosomal recessive	*TUBB1*	chr24:43761303G>A	Norfolk Terrier	Caucasian Shepherd Dog (41; 2.44%) Chesapeake Bay Retriever (138; 3.99%)
469	MDR1 (Multidrug Resistance 1) Medication Sensitivity	Autosomal dominant	*ABCB1*	chr14:13726596_13726599del	>10	Lacy Dog (32; 1.56%)
993	Microphthalmia (Discovered in the Soft-Coated Wheaten Terrier)	Autosomal recessive	*RBP4*	chr28:7830265_7830267del	Irish Soft-Coated Wheaten Terrier	Russell Terrier (239; 6.49%)
69	Neuronal Ceroid Lipofuscinosis 8 (Discovered in the English Setter; NCL8)	Autosomal recessive	*CLN8*	chr37:30874779T>C	English Setter	Gordon Setter (20; 5%)
642	Osteochondrodysplasia (Discovered in the Miniature Poodle)	Autosomal recessive	*SLC13A1*	chr14:60628774_60758561del	Miniature Poodle	Bichon Frise (1069; 2.43%)
[47]	Pituitary-Dependent Hyperadrenocorticism (Discovered in Poodles)	Autosomal dominant	*CRHR1*	chr9:9726624G>A	Poodle	Australian Cattle Dog (982; 2.6%) Great Pyrenees (1985; 11.79%) Maremma Sheepdog (37; 5.41%) Miniature American Shepherd (1476; 2.24%) Portuguese Podengo Pequenos (17; 11.76%) Spanish Water Dog (96; 8.85%)
74	Prekallikrein Deficiency	Autosomal recessive	*KLKB1*	chr16:44501415A>T	Shih-Tzu	Lhasa Apso (243; 3.5%)
365	Primary Lens Luxation (PLL)	Autosomal recessive	*ADAMTS17*	chr3:40782144G>A	>30	Coyote (9; 16.67%) McNab (28; 7.14%) Scottish Terrier (237; 1.05%)
1391	Bardet-Biedl syndrome 2 or Progressive Retinal Atrophy (Discovered in the Shetland Sheepdog; BBS2-PRA)	Autosomal recessive	*BBS2*	chr2:59693737G>C	Shetland Sheepdog	Miniature American Shepherd (1476; 1.36%)
76	Progressive Rod-Cone Degeneration (prcd-PRA)	Autosomal recessive	*PRCD*	chr9:4188663C>T	>50	Bichon Frise (1069; 1.13%) Dalmatian (820; 2.39%) Kai Ken (9; 11.11%) Schnauzer (Miniature) (4638; 1.11%)
552	Van den Ende-Gupta Syndrome (VDEGS)	Autosomal recessive	*SCARF2*	chr26:30237714_30237715del	Wirehaired Fox Terrier	Welsh Terrier (21; 4.76%)
401	Von Willebrand’s Disease, Type 1 (vWD 1)	Autosomal recessive	*VWF*	chr27:38951839G>A	Doberman Pinscher, Kromfohrländer	Boerboel (165; 1.21%) Keeshond (70; 4.29%) Lacy Dog (32; 1.56%) Maltese (2413; 2.03%)

^1^ A reference is listed where a specific OMIA (Online Mendelian Inheritance in Animals; omia.org) variant ID was not available at the time of writing

^2^ CanFam3.1 coordinates

### Mining of electronic medical records (EMRs) establishes clinical significance of disease variants and uncovers evidence for screening relevance on additional breed ancestry backgrounds

Additional evidence supporting variant clinical significance was collected to strengthen the current body of evidence supporting pathogenicity of selected variants, with a special focus on establishing the relevance of unexpected variant findings (variants not previously reported to be present in the breed based on the scientific literature). Medical records were available for a significant proportion of dogs of the genotyped cohort (43.5%; N = 458,433) that had attended a Banfield Pet Hospital veterinary clinic for consultation or treatment, and additional direct interviews with owners were carried out to obtain supplemental phenotype information where possible. With this approach, we evaluated the phenotypes of 12,028 dogs that were genetically at risk based on their DNA testing result (i.e., dogs with two copies of a presumed recessive disorder or dogs with one copy of a presumed dominant disorder) for one of 49 inherited disease-associated variants (key findings in Table 3; full details by dog in S4 Table). In particular, among the variants evaluated in more than one such genetically at risk dog, we observed complete penetrance (defined as 100% of dogs genetically at risk showing clinical signs of the expected disease) of the following variants: canine leukocyte adhesion deficiency (CLAD) Type III [29], disproportionate short-limbed chondrodysplasia (discovered in the Norwegian Elkhound; *ITGA10*-related; [30]), cone-rod dystrophy 2 (discovered in the Pit Bull Terrier; crd2; [31]), focal non-epidermolytic palmoplantar keratoderma (discovered in the Dogue de Bordeaux; [32]), hemophilia A (discovered in German Shepherd Dog; *F8* p.C548Y variant; [33]), hereditary footpad hyperkeratosis (discovered in the Irish Terrier and Kromfohrländer; [34]), ichthyosis (discovered in the American Bulldog; *NIPAL4*-related; [35]), neuroaxonal dystrophy (discovered in the Rottweiler; *VPS11*-related; [36]), skeletal dysplasia 2 (SD2; [37]), and trapped neutrophil syndrome (TNS; [38]).

**Table 3:** Key EMR (electronic medical record)-based clinical validation findings.

OMIA variant ID	Variant phenotype	Mode of inheritance	Gene	Variant^1^	Proportion of evaluated at risk dogs showing phenotype	Breed(s) phenotype confirmed in
576	Canine Leukocyte Adhesion Deficiency (CLAD), Type III	Autosomal recessive	*FERMT3*	chr18:52835932_52835933insGGCAGCCGTCTT	(3/3) 100%	Mixed Breed
336	Chondrodysplasia, Disproportionate Short-limbed (Discovered in the Norwegian Elkhound; ITGA10-related)	Autosomal recessive	*ITGA10*	chr17:58703935G>A	(2/2) 100%	Mixed Breed
606	Cone-Rod Dystrophy 2 (Discovered in the Pit Bull Terrier; crd2)	Autosomal recessive	*IQCB1*	chr33:25078909_25078910insC	(3/3) 100%	Mixed Breed
527	Cystinuria Type II-A (Discovered in the Australian Cattle Dog)	Autosomal dominant	*SLC3A1*	chr10:46725151_46725156del	(2/3) 67%	Mixed Breed
40	Factor VII Deficiency	Autosomal recessive	*F7*	chr22:60578895G>A	(6/33) 18%	Mixed Breed, Basset Hound
936	Focal Non-Epidermolytic Palmoplantar Keratoderma (Discovered in the Dogue de Bordeaux)	Autosomal recessive	*KRT16*	chr9:[21170012_21170013delinsCGGA;21170030del]	(2/2) 100%	Mixed Breed
100	Hemophilia A (Discovered in the German Shepherd Dog; *F8* p.C548Y)	X-linked recessive	*F8*	chrX:122975611C>T	(2/2) 100%	Mixed Breed
89	Hereditary Footpad Hyperkeratosis (Discovered in the Irish Terrier and Kromfohrländer)	Autosomal recessive	*FAM83G*	chr5:41055619G>C	(3/3) 100%	Mixed Breed
49	Hypocatalasia	Autosomal recessive	*CAT*	chr18:33397548C>T	(4/6) 67%	Mixed Breed, Basset Hound
563	Ichthyosis (Discovered in the American Bulldog; NIPAL4-related)	Autosomal recessive	*NIPAL4*	chr4:52737379del	(13/13) 100%	Mixed Breed
98	Macrothrombocytopenia (Discovered in the Norfolk and Cairn Terrier)	Autosomal recessive	*TUBB1*	chr24:43761303G>A	(2/3) 67%	Chesapeake Bay Retriever
995	Neuroaxonal Dystrophy (Discovered in the Rottweiler; VPS11-related)	Autosomal recessive	*VPS11*	chr5:14777774T>C	(2/2) 100%	Mixed Breed
112	Shar-Pei Autoinflammatory Disease (SPAID)	Autosomal dominant (Incomplete penetrance)	*MTBP*	chr13:19383758G>A	(2/4) 50%	Mixed Breed
78	Skeletal Dysplasia 2 (SD2)	Autosomal recessive	*COL11A2*	chr12:2652874C>G	(3/3) 100%	Mixed Breed
945	Spinocerebellar Ataxia with Myokymia and/or Seizures (KCNJ10-related; SCA)	Autosomal recessive	*KCNJ10*	chr38:22140300C>G	(3/4) 75%	Mixed Breed
478	Trapped Neutrophil Syndrome (TNS)	Autosomal recessive	*VPS13B*	chr13:1412654_1412657del	(2/2) 100%	Mixed Breed

^1^ CanFam3.1 coordinates

Overall, the additional compiled evidence supporting causality of the variants listed above was obtained on diverse genetic backgrounds, with varying identified contributions of ancestry from the original variant discovery breeds (S4 Table). Seventy-two percent of the mixed breed dogs that were clinically evaluated showed evidence of the original variant discovery breed(s) present in their ancestry. With focus on variant findings not previously documented in the scientific literature in specific purebreds, we found plausible evidence (based on genetically at risk dogs showing at least some clinical signs that are known manifestations of the evaluated disease) for the clinical relevance of cystinuria in the French Bulldog, factor VII deficiency in the Basset Hound, hypocatalasia in the Basset Hound, macrothrombocytopenia in the Chesapeake Bay Retriever, multidrug resistance 1 (MDR1) related medication sensitivity in the Siberian Husky, and von Willebrand’s Disease Type 1 (vWD 1) in the Boston Terrier, Cairn Terrier, Pomeranian and Pug. Selected validation observations are presented in more detail in the following sections.

### Inherited catalase deficiency is a notable cause of oral ulcers and periodontal disease

The clinical ramifications of the presumed autosomal recessive *CAT* gene variant associated with hypocatalasia (OMIA variant ID 49;OMIA phene ID 001138–9615; [39]) were not well characterized in the pet population previously, as the disorder has been studied mainly in a laboratory colony of Beagles. Using EMR data, we were able to confirm signs consistent with hypocatalasia with an early age of onset in mixed breed dogs with hound ancestry, as well as in one purebred Basset Hound and a mixed breed dog without recent scent hound ancestry (Tables 3 and S4). Clinical signs of the disorder were fairly consistent across dogs at genetic risk and generally included areas of oral ulceration along with gingival recession with bone loss/necrosis and tooth root exposure, causing pain, loss of teeth, and in some cases prolonged bleeding from the mouth. Oral repairs had a tendency to fail due to friable gingival tissue, and clinical signs were progressive in all pets reviewed. These observations, together with our reports of presence of the hypocatalasia variant in pet Beagles, American and English Foxhounds, Harrier, Treeing Walker Coonhound, and Plott Hounds ([4, 12] and the present study) call for greater awareness of hypocatalasia as a health concern especially in dogs of scent hound ancestry.

### Factor VII deficiency presents as a common subclinical propensity for prolonged blood coagulation

The number of identified breeds harboring the autosomal recessive *F7* gene variant associated with Factor VII deficiency (OMIA variant ID 40; OMIA 000361–9615; [40]) has significantly increased after the introduction of genetic panel screening technologies, with the current total exceeding 25 breeds and breed varieties of diverse ancestry [4, 5, 12]. In the present study, EMRs included clotting test laboratory result values for four dogs (three mixed breed dogs and one Basset Hound) homozygous for the *F7* variant. All had an elevated prothrombin time (PT) and a normal activated partial thromboplastin time (aPTT) as anticipated (S4 Table). One of the mixed breed dogs had additionally received factor VII testing, and the value was reduced as expected at 35% of baseline levels. Two additional mixed breed dogs homozygous for the *F7* variant did not have recorded blood clotting measurements but had sought veterinary care due to episodes of hematochezia and hematemesis. Taken together, we note that all blood clotting measurements from *F7* homozygous dogs available for examination by the authors to date have shown the same pattern of elevated PT but normal aPTT times ([4] and the present study). These observations further strengthen the perception that Factor VII deficiency manifests as a mild propensity for prolonged blood clotting, that typically remains clinically undetected, in any dog genetically at risk, regardless of genetic ancestry.

### Multiple genetic causes of a reduced leg length phenotype in dogs

Related retrogenes on chromosomes 12 (12-FGF4RG; OMIA variant ID 855) and 18 (18-FGF4RG; OMIA variant ID 694) represent common and widespread genetic causes of disproportionate dwarfism in dogs, in the form of a characteristic short-limbed phenotype that has become a hallmark of the breed standard in breeds such as Dachshunds, Corgis, and Basset Hounds (OMIA 000157–9615 and 002542–9615; [17, 41]). The chromosome 12 retrogene 12-FGF4RG has particular health relevance as it also confers increased risk for intervertebral disc disease (IVDD) and is associated with age at time of surgical treatment across mixed breed dogs and all affected breeds [42]. We confirm the ubiquitous nature of the 12-FGF4RG variant as the most common tested variant observed in dogs in the present study, with an allele frequency of 11.4% in mixed breed dogs and 13.9% in the combined purebred dog sample (Table 1) and presence in 89 of the examined breeds or breed varieties at a frequency ≥1% (S3 Table and [28]).

**Fig 4:**
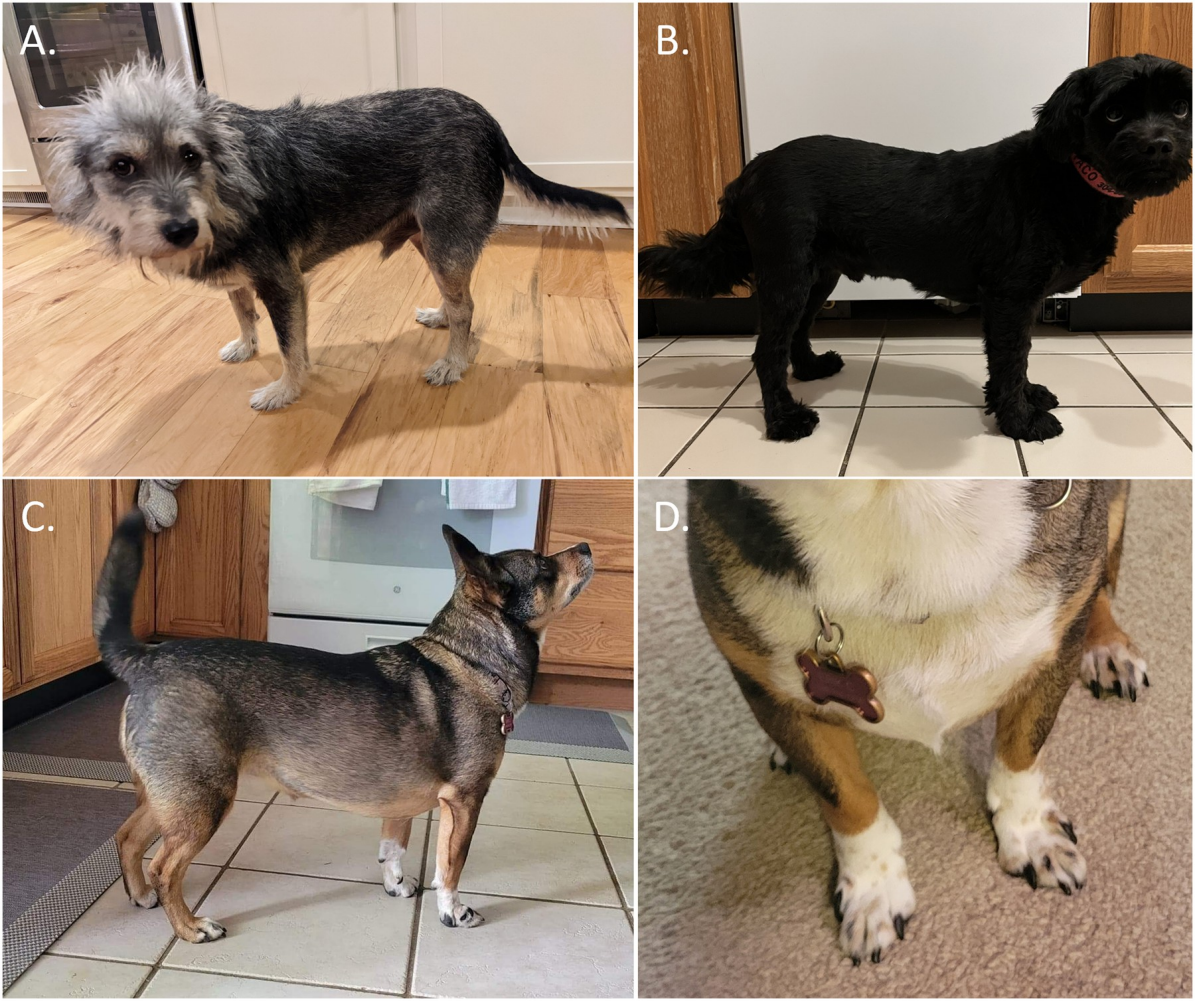
Disproportionate dwarfism phenotypes in mixed breed dogs. A) Skeletal dysplasia 2 (SD2) manifests as a mild phenotype in which the front legs are typically shorter than the hind legs and the body and head are of normal size. B-C) *ITGA10* gene-related disproportionate short-limbed chondrodysplasia manifests as shortened, typically crooked legs with a normal sized head and body and in some cases like D) also as abnormally short outer digits.

In addition to the aforementioned widespread variants for reduced leg length that are inherited in an autosomal dominant or semi-dominant manner, other rare recessive variants have been identified as well. We provide additional confirmation of the phenotype association of two such variants, skeletal dysplasia 2 (SD2; also known as mild disproportionate dwarfism; OMIA variant ID 78; OMIA 001772–9615;) and *ITGA10* gene-related disproportionate short-limbed chondrodysplasia (OMIA variant ID 336; OMIA 001886–9615) [30, 37], on mixed breed ancestry backgrounds. The three dogs homozygous for SD2 were all genotyped as being devoid of any copies of either 12-FGF4RG or 18-FGF4RG and described in their medical history as displaying “all short limbs”, “shortened legs and body longer than dog is tall”, being “cow-hocked” or having a carpal valgus deformity (Fig 4A). Notably, none of the phenotypically evaluated dogs tested as having more than around 5% ancestry contribution from the original variant discovery breed, the Labrador Retriever. The two mixed breed dogs homozygous for the *ITGA10* variant were described as having shortened legs with a normal sized head and body, slight bowing or crookedness of the front legs, and one of them in addition displayed the characteristic abnormally short outer digits previously associated with this phenotype (Fig 4B–4D). Neither of the two showed a notable genetic ancestry contribution (≥1%) from the breeds in which the effects of the *ITGA10* variant have been characterized to date (the Norwegian Elkhound, the Karelian Bear Dog, and the Chinook [4, 30]).

### Genetic and phenotypic heterogeneity underlying canine cystinuria

To date, studies on canine cystinuria have implicated variants in the functional candidate genes *SLC3A1* and *SLC7A9*, encoding amino acid transporters involved in the excretion of cystine and known to explain approximately 70% of human cystinuria cases [23, 43]. Among the putative risk variants examined in the present study were two missense variants (*SLC3A1* p.I192V and p.S698G) previously implicated in a recessive form of cystinuria affecting English and potentially also French Bulldogs and a missense variant *SLC7A9* p.A217T discovered in the heterozygous state in one English Bulldog case (OMIA 000256–9615) [23, 24]. We find that the variants are present in more than 30 breeds of diverse ancestry with some of the highest allele frequencies observed in Bulldog and Mastiff-type breeds (S3 Table). We find that the *SLC3A1* variants coexist in strong linkage disequilibrium (r^2^ > 0.99) in many, but not all, breeds they are present in, laying the foundation for further efforts to pinpoint the risk-conferring genetic variation at the locus. Several non-mastiff breeds have a high frequency of the *SLC7A9* variant alone (e.g., Golden Retriever, Swedish Vallhund, and English Cocker Spaniel; allele frequencies 12–26%) calling into question if the variant is a risk factor for cystinuria on its own. Moreover, very few (between 0–1.5% depending on the breed) English or French Bulldogs, American Staffordshire Terriers, Golden Retrievers or mixed breed dogs at putative genetic risk due to their *SLC3A1* and *SLC7A9* genotypes showed clinical signs (EMR-documented cystic calculi, crystalluria, hematuria, urolithiasis, nephrolithiasis, urinary tract infection or cystitis) supporting a diagnosis of cystinuria.

Another variant in the *SLC3A1* gene (p.T366_T367del; OMIA variant ID 527; OMIA 001879–9615) was proposed to cause an autosomal dominant form of the disease in Australian Cattle Dogs, with a more severe phenotype in homozygous dogs [43]. We evaluated three mixed breed dogs, two homozygous and one heterozygous for *SLC3A1* p.T366_T367del and confirm that the variant was associated with a fairly severe morbidity with the examined homozygous patients showing their first signs of obstruction by 7 months of age. Both affected dogs passed or were euthanized before the age of two years. The third dog heterozygous for the variant and with available EMR data has not returned to the clinic since approximately nine months of age but did not show any significant clinical diagnoses except for submissive urination at the last visit.

### Additional evidence for specific highly penetrant and clinically relevant canine disease variants

In addition, the EMRs provided further evidence supporting the causality and high penetrance of recessive variants previously associated with cone-rod dystrophy 2 (crd2; OMIA variant ID 606; OMIA 001675–9615 [31]), spinocerebellar ataxia with myokymia and/or seizures (SCA; *KCNJ10*-related; OMIA variant ID 945; OMIA 002089–9615 [44]), and both the Golden Retriever (*PNPLA1*-related; OMIA variant ID 616; OMIA 001588–9615 [45]) and American Bulldog (*NIPAL4*-related; OMIA variant ID 563; OMIA 001980–9615 [35]) ichthyoses. In the three mixed breed dogs evaluated due to their expected genetic predisposition to develop crd2, the retinal condition appears to show a particularly early age of onset, with overt clinical signs beginning at 3–5 months of age. Evaluated dogs were all reported to show signs of vision loss such as walking into objects, loss of the menace response, and clumsiness. All were apparently blind on exam by the age of one year. Similarly, the genetic variant associated with SCA [44] seemed to show a high penetrance in mixed breed dogs with characteristic clinical signs such as dizziness, ataxia, weakness in the hind limbs (particularly in the morning hours), seizures, and even aggression and blindness reported in three of four evaluated dogs. The fourth patient was described in the EMR as having an episode of hindlimb lameness, which may be suggestive of SCA, but follow up examination/diagnostics were not available.

In contrast to the severe signs in the above disorders, while the forms of ichthyosis originally characterized in Golden Retrievers and American Bulldogs [35, 45] are not as life threatening, they were correlated with the anticipated clinical signs in all dogs genetically at risk evaluated. These dogs presented with flaky, scaly skin as puppies, with some owners reporting seeing flaking from birth and the majority of owners noticing ‘dry skin’ at the first puppy exam (approximately eight weeks). In many cases, these dogs did present with multiple dermatologic episodes throughout the timeframe evaluated, showing secondary infections, pyoderma, alopecia, and otitis along with generalized scaling.

## Discussion

With the present study we aimed to expand knowledge on the distribution, genetic prevalence, and clinical relevance of an unprecedented number of canine disease-associated variants in a dog population of unparalleled size, focusing on previously identified polymorphisms that follow a Mendelian mode of inheritance. We further explored the link between genetic inbreeding levels measured on an individual dog level and Mendelian disease risk.

In line with our previous investigation of 152 disease-associated variants in around 100,000 dogs [12], we found that most tested variants are shared by both the mixed breed and the combined purebred population. The most notable effect of a 10-fold increase in study population size and the addition of nearly 100 tested variants to the screening panel is that the proportion of variants that are found in both the mixed and purebred group has more than doubled compared to our previous study (from 41% to 88%) while fewer variants remain exclusive to either subgroup. This observation is explained in part by our conscious decision to include several variants with inconclusive evidence of causality beyond the original discovery breed for genotyping in the present study (e.g., [22, 23, 25, 46, 47]), but it is also likely that a larger mixed breed sample captures a wider variety of breed backgrounds and disease alleles segregating in the population. The proportion of variants not encountered in the study sample remained comparable to our previous study at approximately 17%, despite the increased number of tested variants. The collective group of non-observed variants consisted of several X-linked disease variants (e.g., hemophilia A and B; S1 Table), and variants originally characterized as private to specific cases or family lines (e.g., Alexander disease in Labrador Retriever, osteochondromatosis in American Staffordshire Terrier and congenital eye malformations in Golden Retriever [48–50]) and we consequently confirmed the absence of these variants from the broader canine population. Another notable observation compared to our prior canine disease variant screening studies is that with the increase of the number of tested variants to 250, it is now more common than not for a dog to carry at least one variant potentially influencing disease risk (57.2% of dogs). This estimate highlights how management of inherited disease in breeding programs of purebred populations requires thorough screening of known disease-associated variants with a particular focus on the variants that are most relevant within each breed, combined with simultaneous consideration of genetic diversity and the viability of the gene pool in breeding selections.

We, in collaboration with other investigators, have previously shown that breeds with higher-than-average inbreeding levels required greater amounts of veterinary care on average [51], and that purebred dogs as a group are more likely than mixed breed dogs to be homozygous for a common recessive disease-associated variant [12]. In the present study, we have for the first time been able to evaluate the correlation between a molecular measure of genome-wide inbreeding and overall Mendelian disease variant load on an individual level. We most notably observed a relationship, albeit one with a small correlation coefficient, between genome-wide reduction in genetic diversity and an increased risk of homozygosity for multiple autosomal/recessive disease-associated variants in dogs. This finding provides a direct measurement-based example of how increased genomic homozygosity, e.g. as a consequence of the mating of close relatives in a closed gene pool, could contribute to driving inbreeding depression through the accumulation of deleterious recessives. Notably, mixed breed dogs as a group are no exception to the observed correlation between diversity levels and disease-associated variants. While it is theoretically likely that disease-associated variants are maintained in the mixed breed population through random breeding with frequencies varying over time due to random genetic drift, matings between closely related individuals are known to occur in settings such as puppy mills. This explains the very broad spectrum of diversity levels observed in mixed breed dogs and emphasizes the utility of recessive disease testing also in this group in a veterinary clinical setting.

While we show that disease-associated variants are collectively common in dogs, we also note that the majority of the studied specific variants have a frequency of <1% in the population, making it difficult for any individual veterinary clinician to recognize and maintain proficiency with the broad spectrum of characterized inherited disorders. This stresses the need for continuing medical education and veterinary specialists trained within the field of genetic counseling, while highlighting the value of broad diagnostic screening technologies. Concerns regarding their appropriate application in direct-to-consumer settings and veterinary medicine have nevertheless been raised along with a call for industry standards related to testing methodology and reporting [52]. Collective efforts to establish best practices for the field are underway and will undoubtedly be crucial for leveraging the potential that genetic testing has to support individual and breed population health, well-being, and welfare in dogs [53, 54].

The most common disease-associated variants observed in the present study expectedly included some known genetically widespread variants that are also frequently requested targets for genetic screening, such as DM, CEA, prcd-PRA, HUU, exercise-induced collapse (EIC), MDR1 (multidrug resistance 1) medication sensitivity, and von Willebrand’s disease type 1 (vWD 1) [18–21, 55–57]. Despite extensive genetic testing for these variants, challenges in determining and understanding their penetrance and expressivity in different breed backgrounds partially persist, as exemplified by phenotype studies of dogs genetically at risk of DM, CEA, and cone-rod dystrophy (cord1-PRA/crd4) [58–60]. For these and other variants, there are potentially unidentified genetic risk and age of onset modifiers that blur a clear Mendelian inheritance pattern. It can also be particularly difficult to interpret what role in the regulation of disease onset is played by variants originally published based on study of a subset of the canine population, or with limited functional evidence supporting causality. Within the scope of this study, we present selected clinical validation follow-up studies that support the relevance of specific variants on diverse genetic ancestry backgrounds, while laying the foundation for future prospective reports addressing additional late onset conditions as our cohort of tested dogs ages. We show that some variants previously associated with bald thigh syndrome in sighthounds, dilated cardiomyopathy (DCM) in Doberman Pinschers, and Bulldog-type cystinuria are very common in the broader canine population and may represent non-causal markers in themselves. The lack of correlation between genotype and phenotype for several variants (such as ones included in our investigation but not yet assigned an OMIA variant ID) is not surprising given that their original discoverers have acknowledged uncertainty regarding causality. We caution against making conclusive health care or breeding related decisions based on genetic testing for such variants alone in dogs of mixed breed ancestry or in other pure breeds until further studies are conducted. Investigations of the impact of putative genetic risk factors in dogs representing a diverse genetic ancestry background are essential for establishing variant causality and broader clinical relevance of genetic testing. Among other findings, we provide evidence suggesting in particular that the specific variants previously associated with canine leukocyte adhesion deficiency type III, *ITGA10*-related chondrodysplasia, hypocatalasia, factor VII deficiency, cone-rod dystrophy 2, *KCNJ10*-related spinocerebellar ataxia, both *PNPLA1* and *NIPAL4*-related ichthyoses, focal non-epidermolytic palmoplantar keratoderma, hemophilia A, *FAM83G*-related hereditary footpad hyperkeratosis, *VPS11-*related neuroaxonal dystrophy, skeletal dysplasia 2 or trapped neutrophil syndrome have high or complete penetrance across breed ancestry backgrounds, which suggests that these variants always bear relevance when present in a dog. Taken together, the body of evidence we have collected contributes to establishing genetic counseling and veterinary care guidelines related to canine disease-associated variants. Breeding decisions informed by observations collected across the broad dog population are critical both to avoid producing offspring at risk of a disease and to ensure that dogs carrying a non-causal variant are not unnecessarily excluded from breeding programs.

The present study represents the largest cohort of dogs and the most extensive set of Mendelian disease-associated variants examined in a single study to date. The study sample is broadly representative of the breed composition in the canine pet population due to ascertainment both via direct-to-consumer genetic testing (pursued in the vast majority of cases out of curiosity regarding breed ancestry, or to a lesser extent, with the aim to select appropriate mating pairs and meet genetic health screening requirements) and DNA sampling during primary veterinary care which was largely carried out without prior suspicion of patient susceptibility for a specific genetic disease. We acknowledge as limitations that the cohort does not account for the population of dogs that does not undergo genetic testing for diverse reasons (such as lack of owner interest, awareness or financial means), that the relatedness status of dogs remained unknown to us and that the sample is biased towards dogs from the United States. However, the study does include representation of more than 250 different breeds or breed varieties and more than 150 countries or geographic territories, and we are therefore confident that the generated dataset is a valuable resource for the community that enables breed health committees and researchers to examine variant frequencies and genetic diversity levels by breed and region in more detail. We do acknowledge that the presented heterozygosity estimates are sensitive to the specific SNP-marker content that they have been produced with and that the most exact genome-wide variation data is derived from broader sequencing efforts. Large cohorts with both genotyping data and detailed medical history available are nonetheless an invaluable resource for studying disease etiology and epidemiology, and while we note that the group of dogs with electronic health records accessible to us was of a young median age (1.17 years at the last recorded clinic visit), we lay a solid foundation for a prospective study. When interpreting the clinical validation outcomes and estimates of variant penetrance, it should nonetheless be noted that some genetically at risk dogs may only go on to develop signs of disease later in life or might have been lacking the specific laboratory examination required for a definitive diagnosis. This is especially exemplified by our examination of *SLC3A1* and *SLC7A9* variants in cystinuria, where we cannot exclude the possibility that the studied missense variants represent modifiers or linked markers predictive of disease risk on some breed backgrounds such as Bulldog ancestry due to undiagnosed signs of cystinuria in the EMRs, gender-specific differences in manifestation, or later onset of disease. Moreover, our investigations of presumed autosomal recessive disorders focused on dogs homozygous for the alternative allele within the scope of the present study and potential predisposing effects in heterozygous carrier dogs therefore warrant future examination. While our cohort provides the best available insight into the Mendelian disease variant heritage of the general dog population, we specifically acknowledge that our definition of a “purebred” (a genetically uniform group of dogs with a consistent, predictable conformational and behavioral type) also included dogs that did not have a documented pedigree as defined by a formal breed registry. Moreover, as for any diagnostic test and even with the strictest quality control measures, some potential for individual spurious false positive test results exists without follow up with a secondary genotyping technology such as sequencing across the whole cohort. We have focused on presenting only the most relevant disease variant findings (>1% frequency and more than one alternative allele heterozygous/homozygous dog observed) in additional breeds for these reasons.

In conclusion, we report that it is relatively common for a dog to be at least heterozygous for one of the many known putative canine disease variants, while the majority of individual variants are very rare in the overall population. While many variants are enriched in specific breeds and absent from others, we conclude that health issues caused by Mendelian disease-associated variants are broadly shared across both mixed and purebred populations, as we find that several variants cause similar signs of disease independently of breed ancestry. Understanding of the clinical relevance of variants that are already targets of commercial genetic tests represents an important advance towards creating a variant significance classification system for dogs and contributes to the enablement of precision medicine. As more disease-associated variants are identified and screened in the future, it is likely to become inevitably clear that all dogs carry a number of deleterious recessive alleles. Maintenance of population diversity through sustainable breeding selections, outcrossing programs, and avoiding the overuse of specific popular sires represents a simple remedy that keeps breeds viable by counteracting the augmentation of recessive disease alleles.

## Materials and methods

### Ethics statement

The study protocol was reviewed and approved by the Institutional Review Board at the Waltham Petcare Science Institute, Mars Petcare, UK. Canine DNA was obtained as owner submitted, non-invasive cheek swab samples or collected by certified veterinary clinics as either cheek swab or blood samples in accordance with international standards for animal care and research. All owners provided written consent for the use of their dog’s DNA sample in scientific research and separate consent was collected for the use of clinical phenotype data where it was available.

### Dryad DOI

https://doi.org/10.5061/dryad.tdz08kq3j

### Study sample

A total of 1,054,293 dogs (811,628 mixed breed and 242,665 purebred dogs) were successfully genotyped as a part of this study. All DNA samples were voluntarily submitted for commercial genetic testing (Wisdom Panel, MyDogDNA, and Optimal Selection Canine genetic screening products) by Wisdom Panel (Portland, OR, USA) between November, 2019 and August, 2021. The relatedness status of dogs was unknown at the time of sample submission. The study cohort represented a subset of the total more than 3.5 million dogs of varying ancestry genetically tested at Wisdom Panel since the service launch. A total of 1,086,817 samples were first selected based on having been genotyped on the largest available cross-compatible microarray technology platform and 32,524 (3%) of them were excluded due to not passing our routine quality control metric for genotyping (sample-specific call rate >0.97%). The country of origin of each dog was defined as the country where the sample was submitted for genetic analysis, unless specific information stating otherwise was reported by the owner. A total of 160 countries or autonomous regions were represented in the dataset (96 regions with >5 dogs). The vast majority of dogs (93.9%) were from the United States, with other notable subgroups being dogs from the United Kingdom (2.5%), Germany (1.2%), France (0.5%), Australia (0.3%), Finland (0.2%), and Canada (0.2%).

As one major motivation for clients to pursue Wisdom Panel genetic testing is gaining insight into their dog’s breed ancestry, the purebred status of a dog was either not known or considered prior to genotyping. Breed assignment was based on comparison to a reference panel of over 21,000 dogs of known ancestry from more than 50 countries and ascertained using the BCSYS Local Ancestry Classifier algorithm [61]. For the purposes of this study, a dog was considered “purebred” if its genetic testing results indicated at least 7 of 8 great-grandparents being purebreds of the same breed. Notably, we did not strive to use a definition of “purebred dog” synonymous with the term “pedigreed dog” in terms of eligibility for registration with a recognized kennel club or breed registry. The purebred cohort (S2 Table) consisted of 263 different breeds or breed varieties (218 breeds represented by >5 dogs) and 34 samples from wild canids (gray wolves, dingos, and coyotes). Breeds contributing more than 2% of individuals of the overall study sample were: American Staffordshire Terrier (17.6%), Labrador Retriever (6.9%), German Shepherd Dog (6.4%), French Bulldog (5.4%), Golden Retriever (5.3%), Siberian Husky (3.7%), Yorkshire Terrier (3.4%), Shih Tzu (3.1%), Border Collie (2.8%), Pomeranian (2.2%), Beagle (2.2%), Pug (2.1%), Chihuahua (2.2%), and Standard Bulldog (2.0%).

### Genotyping

Genotyping of 250 disease-associated variants and 1,877 genome-wide representative and cross-microarray platform version available SNP markers used for genetic heterozygosity evaluation was carried out according to manufacturer-recommended standard protocols on a custom-designed Illumina Infinium XT microarray (Illumina, Inc., San Diego, CA, USA). Protocols for microarray design, validation, and data quality control have previously been described in detail [4, 12, 62]. Samples with a genotyping call rate <97% were excluded from the present study. The heterozygosity level was calculated for each dog as the ratio of heterozygous SNP genotypes out of the total number of non-missing SNP genotypes in the set of 1,877 neutral markers (average marker spacing = 1,155,501 bp). As validation of marker set utility, we have previously shown that breed median heterozygosity values measured with the same approach correlate well (Pearson r = -0.89, P < 0.0001; based on 19 breeds) with the coefficient of inbreeding (F) values calculated using Illumina canine HD genotyping array data (173 K SNPs) for the same breeds [51]. Moreover, this SNP platform has also been successfully utilized to elucidate breed genetic relationships, effective population size, and population differentiation [63–66]. Mendelian variants were selected for analysis based on pre-existing evidence implicating the variants in the etiology of canine inherited disorders, either as summarized in the Online Mendelian Inheritance in Animals (OMIA) database (https://www.omia.org/. Last accessed March 9^th^, 2022) or based on a literature review. No genotype information became available for variants outside of the ones specifically included in the design. A small number of more recently characterized genetic variants (N = 9; S1 Table) were only available on a later updated version of the custom microarray and they were consequently genotyped in a subset of dogs (N = 46,062). The generated genotypes for disease-associated variants, and sample metadata, is available at https://doi.org/10.5061/dryad.tdz08kq3j [28].

### Statistical analyses

All statistical modeling was carried out in Minitab version 19.2020.1 statistical analysis software. Standard non-parametric tests were used for comparisons of heterozygosity levels between groups, as the variable was non-normally distributed in both mixed breed and purebred dogs (Kolmogorov-Smirnov KS_mixed breed_ = 0.207; KS_purebred_ = 0.048; P < 0.01). The Mann-Whitney U test was used for comparisons of two groups (e.g, mixed breed and purebred dogs), and the Spearman rank correlation test was used to evaluate the relationship between heterozygosity level and N disease-associated variants carried in the heterozygous or homozygous state. For the correlation analysis, the small number of dogs carrying >5 variants (N = 35 mixed breed and purebred in total) were combined with dogs carrying 5 variants to obtain informative subgroup sizes of ≥20 dogs.

### Clinical data evaluation

The following approach was taken to explore the relationship between genetic variants and their respective expected associated phenotypes. Manual cross-linking of sample ID number, with verification through any additional identifying information available, between the full genotyped cohort and electronic medical records (EMRs) of dogs enrolled for Optimum Wellness Plans at Banfield Pet Hospital clinics during the years 2018–2021 revealed 458,433 dogs for further consideration. EMR data including diagnoses with standardized ailment codes was pulled for the aforementioned dogs, with personal identifiable information removed. The detailed medical notes for dogs at risk of a certain disorder as suggested by their genetic testing results (dogs with two copies of a presumed recessive disorder, or dogs with one copy of a presumed dominant disorder) were manually reviewed by a veterinary clinician to obtain confirmation or refutal of the expected clinical phenotypes. Additional unstructured interviews between dog owners and a Wisdom Panel veterinarian or geneticist were used as a supplementary tool to collect phenotype information, previous clinical examination records or photographic evidence.

## Supporting information

S1 Table Allele and genotype frequencies, and prevalence ranks for 250 screened variants in the full study sample. (XLSX)

S2 Table Study sample composition and descriptive heterozygosity statistics by breed. (XLSX)

S3 Table Summary of disease allele frequencies across breeds. (XLSX)

S4 Table Clinical evaluation outcomes by disease and phenotype details by dog. (XLSX)
